# Medical Association of Ireland

**Published:** 1840-07

**Authors:** 


### MEDICAL ASSOCIATION OF IRELAND.

The First Anniversary Meeting of the Association was held in Dublin, on Wednesday, the 27th ultimo, and was very numerously attended by representatives of the various local medical associations, and by individual members from every part of Ireland. The President, Richard Carmichael, Esq. was in the chair, and opened the meeting by an excellent address. The Report of the Council was then read by the Secretary, Dr. Maunsell, and was unanimously adopted and ordered to be printed. The meeting was afterwards addressed by Dr. O'Beirne, Dr. Morrison, Dr. Jacob, Dr. Colvan, Dr. O'Grady, Dr. Kingsley, Dr. John Jacob, Dr. O'Brien, Dr. Benson, Dr. Purcell, Dr. Ferguson, Dr. Jagoe, Dr. Hargrave, Dr. Cranfield, Dr. Macdonnel, Dr. Cane, Dr. M'Mullen, Dr. Williams, Dr. Healy, Dr. O'Reardon, Sir James Murray, and others, at very considerable length and with much eloquence. We regret that our space will only permit us to give the principal resolutions adopted by the meeting, which was most interesting, and cannot fail to be of the greatest importance to the cause of medical reform.

*Resolutions passed at the Meeting.*

1. That this association, in its collective capacity, is unconnected with any college, corporation, or body, and that it is designed to advance the interests of no party whatsoever, but solely and singly to promote the welfare of the public and of the members of the medical profession, without any difference or distinction.

2. That the permanent continuance of a body capable of advising and protecting medical men in the discharge of their duties and maintenance of their rights, suited to watch over professional interests, and to be the means of communicating between the profession and the government, is highly desirable.

3. That we constitute the council of this association as the organ of communication, on all matters concerning the interests of the medical profession, between its members and the government.

4. That the objects of the association are: 1. To form a society for the protection of medical practitioners in all their just and legal rights. 2. To seek for a legislative enactment giving a permanent constitution to the profession, and directing a competent and uniform standard of education and an equality of privileges for all persons who shall, in future, be permitted to practise medicine throughout the empire. 3. To secure for the public, in future, the services of a scientific apothecary, who shall be protected in the exercise of his profession, and not engage in the practice of medicine.

5. That the council be directed to prepare a petition to parliament, for the enactment of a measure which shall provide for the regulation and control of the medical charities; also praying that adequate funds shall be provided for their support.

6. That the council be directed to prepare petitions to parliament for suitable remuneration to medical men when called upon to perform public services in courts of justice.

7. That the council be instructed to prepare petitions to parliament, praying for attention to the neglected subject of medical police, and for encouragement to medical men disposed to engage in the investigation of all matters concerning the public health; and that copies of these different petitions shall be forwarded to each local secretary, in order to procure signatures in all parts of Ireland.

8 That the following plan of general medical reform shall be supported by this association: [See No. I. of "Plans of General Medical Reform," *infra*, p.297.]

Mr. Carmichael was reelected president; Dr. Macdonnel, Treasurer; and Dr. Maunsell, Secretary; and it was fixed that the future meetings of the association should always be held on the last Wednesday in May. It gives us great pleasure to add that the first-named gentleman has, since his reelection, placed £500 at the disposal of the council of the association, for the purpose of carrying out the objects of medical reform. This act of Mr. Carmichael is worthy of his high character and distinguished reputation, and cannot fail to influence most favorably the great cause of which he is so consistent and so enlightened an advocate. We doubt not but this example will be followed by other members of the profession; and we are disposed to call on the medical reformers of England to testify their sincerity and zeal in the same cause by a general contribution, either in small annual sums or in more considerable donations at once, to be exclusively devoted to the furtherance of the grand object. It has rarely happened that so great a measure as we are contending for has been compassed without much expenditure of money as well as of time and trouble; and when we regard the formidable obstacles to be surmounted ere we reach our aim, and the vital importance of reaching that aim, it cannot be supposed that any one who is really sincere will hesitate for a moment in giving this proof of his sincerity, if it can be shown that it is essential to the success of medical reform.

---

